# The Role of Shared Decision-Making in Promoting Family Participation in Treatment for Adolescents and Young Adults with First-Episode Psychosis

**DOI:** 10.1007/s10597-024-01363-7

**Published:** 2024-12-07

**Authors:** Nicole L. Davies, Paige E. Cervantes, Mary C. Acri, Kimberly E. Hoagwood

**Affiliations:** Department of Child and Adolescent Psychiatry, NYU Langone Health, New York, NY USA

**Keywords:** Shared decision-making, Family participation, Caregiving, First-episode psychosis, Engagement

## Abstract

We aimed to examine the role of shared decision-making (SDM) in family participation in the treatment of adolescents and young adults with first-episode psychosis (FEP). Based on responses of 144 family members of OnTrackNY (OTNY) participants, we divided the sample into low participators and high participators. We calculated the total SDM score for each participant by summing the ratings across items inquiring about SDM and assessed the extent to which loved ones encouraged family participation in their care. Our results indicated that the level of loved ones’ encouragement was significantly related to family participation. When controlling for loved ones’ encouragement, we found that the total SDM score was significantly higher in the high participator group. These findings suggest that SDM may be influenced by loved ones’ attitudes towards family involvement in treatment and SDM may play a role in promoting family participation in care for individuals with FEP.

## Introduction

Approximately 100,000 adolescents and young adults experience first-episode psychosis (FEP) annually (Kirkbride et al., 2012), and FEP was ranked the 11th most common cause of disability worldwide in 2013 (Vos et al., 2015), yet recovery rates have remained stagnant (Fusar-Poli et al., 2017). Engagement in long-term treatment can improve overall symptoms and functioning and decrease relapse (Robson & Greenwood, 2022; Stowkowy et al., 2012). However, nearly one-third of individuals with FEP disengage from services, and this is even more common if they enter an FEP program without family support (Doyle et al., 2014; Franco Mascayano et al., 2021). Further highlighting the need for family involvement in the care process, living without or away from family during FEP treatment significantly predicts service disengagement (Schimmelmann et al., 2006). According to the National Institute of Health, low engagement and retention in services pose considerable threats to the effectiveness of evidence-based interventions, such as those used for treating FEP (Ingoldsby, 2010). Thus, identifying strategies to facilitate family engagement could potentially improve FEP treatment retention and bridge the gap between mental health needs and service use, ultimately decreasing the relapse rate of adolescents and young adults.

Involving family members in the treatment process can improve the treatment experience for both the patient experiencing FEP and their family member (Claxton et al., 2017). Family members are often engaged in a long-term caretaking role, and often are financially responsible and experience emotional distress surrounding their loved one’s condition and care (Interdepartmental Serious Mental Illness Coordinating Committee, 2017), emphasizing the importance of their involvement in the treatment process. The shared decision-making (SDM) model is an approach with the potential to enhance family member involvement due to its supportive clinical culture that prioritizes open communication and collaboration. The SDM model allows clinicians, patients, and families to work together to make care decisions based on both clinical knowledge and participant preferences and values (Elwyn et al., 2010). This healthcare approach falls under the broader concept of patient-centered care, which has gained increasing attention in the healthcare field over the past 10 years and works to personalize each care approach to take patients’ values and preferences into account (Engle et al., 2021). SDM interventions have demonstrated promising outcomes for those experiencing severe mental health symptoms such as improving treatment adherence and treatment-related empowerment (Fiorillo et al., 2020; Stovell et al., 2016). Young adults being treated for severe mental illnesses may especially benefit from participation in SDM due to engagement challenges (Thomas et al., 2021; Dixon et al., 2016). Further, previous studies signal that patients with mental health disorders both want and have the capability of participating in SDM (Zisman-Ilani et al., 2017) and indicate that SDM is a patient engagement facilitator (Lucksted et al., 2015). In response to such findings surrounding the use of SDM in patients with mental health disorders, the American Psychiatric Association and the Substance Abuse and Mental Health Services Administration support the acknowledgment of SDM as a critical care practice (Zisman-Ilani et al., 2021a, 2021b).

While previous work targets SDM and the decisions of patients to involve family members in their care (Dixon et al., 2014), there is a need to explore the relationship between SDM and family engagement in mental health care. This lack of family engagement focus is evident in family members noting their involvement is limited to practical caregiving tasks due to a lack of engagement access and support (Huang et al., 2021). Additionally, caregiving needs at large are not adequately targeted by the mental health system (McNeil, 2012) and the focus on patient empowerment should be applied to family members to encourage them to take an active role in their loved one’s journey seeking services (Deegan, 2007). Given that young adults experiencing severe mental health symptoms may need support to make care decisions, it is critical to consider the role of family members in the SDM approach (Karnieli-Miller et al., 2012) and include family member perspectives in our understanding of patient-centered care (McNeil, 2012).

In this study, we aimed to analyze the role of SDM in promoting family participation in the treatment of youth and young adults with FEP. To conduct this analysis, we used questions capturing family members’ perceptions of SDM and their experiences receiving services by distributing a survey to family members of adolescents and young adults served by OnTrackNY (OTNY), NY state’s treatment program for FEP for individuals between the ages of 16 and 30 (Bello et al., 2017). Family participation in OTNY includes contacts between the family and the multidisciplinary care team and the use of family services such as participation in family psychoeducation and group meetings. Questions targeting SDM were developed based on the work of Deegan and Drake (2006). This survey was conducted in partnership with OTNY leadership and the 23 OTNY teams across NYS. For the purpose of this paper, we are using the terminology of family members to include all different individuals who may be caring for a young adult experiencing FEP.

## Method

### Procedure

In December 2020, letters inviting family members to respond to our survey were sent in both English and Spanish to all 23 OTNY sites for providers and staff to distribute to family members. This article reports on early results from a larger study still underway (Hoagwood, PI for P50 NIMH ALACRITY Center). The corresponding letter introduced the survey, described its purpose, and stated that responses were anonymous, participation was voluntary, and that no identifying information would be collected. Participants had the option of completing the survey online through REDCap, by phone with a member of the research team, or on a hard copy provided with the letter to be returned by mail in a pre-stamped, pre-addressed envelope. The survey was also distributed in Mandarin if requested. Questions required either a short answer response or a response on a Likert scale and assessed five domains: (1) overall perceptions of OTNY, (2) barriers to participation in OTNY, (3) communication with OTNY teams and preferences, (4) changes in participation over time and reasons for shifts in participation, and (5) helpful materials and resources. Our study did not meet the definition of human subjects’ research and was determined to be a quality improvement project through NYU’s self-certification process. For the purpose of this study, we focused on questions asking about family members’ perceived level of participation in OTNY, relative’s preference for involvement, and OTNY use of specific SDM components.

The items inquiring about level of family participation and loved one’s encouragement of family involvement were rated on a 5-point Likert scale and dichotomized. Scores were dichotomized to enable stronger comparisons with the small sample size at hand. Ratings of 1–3 indicated low family participation and no loved one encouragement of family involvement and 4–5 indicated high participation and encouragement of family involvement. Categories were based on clinical judgement, as neutral participation (i.e., rating of 3) was not considered equivalent to endorsing high participation. Five items inquired about OTNY use of SDM and were rated on a 4-point Likert scale and are listed in Table 1. Ratings across these items were summed to calculate a total SDM score. Following this calculation, SDM item responses were also dichotomized (i.e., 1–2 = No, 3–4 = Yes).

**Table 1:** Differences among high and low participation family member groups

	Low participation group (*N* = 45)	High participation group (*N* = 78)	Full sample *(N* = 123)
Perceived participation level			
Very low *N* (%)	3 (2.4)	–	3 (2.4)
Low *N*(%)	9 (7.3)	–	9 (7.3)
Neutral *N* (%)	33 (26.8)	–	33 (26.8)
High *N* (%)	–	51 (41.5)	51 (41.5)
Very high *N* (%)	–	27 (22.0)	27 (22.0)
Loved one encourages family participation*			
Strongly disagree *N* (%)	3 (2.4)	4 (5.1)	7 (5.7)
Disagree *N* (%)	5 (11.1)	2 (2.6)	7 (5.7)
Neutral *N* (%)	18 (40.0)	24 (30.8)	42 (34.1)
*Does not encourage participation N (%)*	*26 (57.7)*	*30 (38.5)*	*56 (45.5)*
Agree *N* (%)	12 (26.7)	31 (39.7)	43 (35.0)
Strongly agree *N* (%)	7 (15.6)	17 (21.8)	24 (19.5)
*Encourages participation N(%)*	*19 (42.2)*	*48 (61.5)*	*67 (54.5)*
SDM Ratings			
*Item 1*: The team emphasizes to families that there is more than one way to deal with an identified problem and asks about participant and loved ones' preferences.*			
Definitely no/no *N* (%)	7 (15.6)	1 (1.3)	8 (6.5)
Yes/Definitely yes *N* (%)	38 (84.4)	77 (98.7)	115 (93.5)
*Item 2*: The team explains the pros and cons of options, including taking 'no action.'*			
Definitely no/no *N* (%)	7 (15.6)	3 (3.8)	10 (8.1)
Yes/Definitely yes *N* (%)	38 (84.4)	75 (96.2)	113 (91.9)
*Item 3*: The team explores your expectations or ideas about how problems are to be managed.*			
Definitely no/no *N* (%)	5 (11.1)	0 (0.0)	5 (4.1)
Yes/definitely yes *N* (%)	40 (88.9)	78 (100)	118 (95.9)
*Item 4*: The team asks how much you want to be involved and respects or values your involvement			
Definitely no/no *N* (%)	4 (8.9)	2 (2.6)	6 (4.9)
Yes/definitely yes *N* (%)	41(91.1)	76 (97.4)	117 (95.1)
*Item 5:* The team understands, appreciates, and respects your values and presents you with options that are in line with them.*			
Definitely no/no *N* (%)	3 (2.4)	0 (0.0)	3 (2.4)
Yes/definitely yes *N* (%)	42 (93.3)	78 (100)	120 (97.6)
SDM total score*			
M(SD)	11.6 (3.3)	13.9 (1.7)	13.1 (2.6)
Range	1–15	8–15	1–15

Italic values highlight the dichotomized variables

*Statistically significant difference between groups

### Participants

Between December 2020 and June 2021, 736 surveys were distributed by the 23 OTNY sites, and 144 family members of adolescents and young adults served by OTNY completed the survey, representing a 19.57% response rate. Twenty-one responses (14.6%) were missing data on items related to key variables, including participation, encouragement, and SDM total score, and were removed from the sample. Analysis was conducted on the remaining 123 surveys.

### Statistical Analyses

We evaluated the association between the perceived level of family participation in care and the perceived level of encouragement of family participation by the loved one enrolled in OTNY using a chi-square test, following standard practice. We then examined the association between perceived use of SDM strategies and family participation level. Because family participation likely varies by loved one preference, which is largely out of the control of the provider regardless of use of SDM, we used an analysis of covariance, with level of family participation as the independent variable, SDM total score as the dependent variable, and level of loved one’s encouragement of family participation as the covariate. Differences in endorsements on the items included in the SDM scale were also examined across High and Low Participation groups. Due to a violation of chi-square test assumptions (i.e., expected cell count), we used Fisher’s Exact tests for item-level analyses.

## Results

### Sample Characteristics

We found no significant differences between groups in sociodemographic characteristics, as displayed in Table 2. However, we noted differences in Black family participants in the Low (26.7%) versus High (19.2%) participation group and white family participants in the Low (31%) versus High (41%) participation group. In the High Participation group, 27 participants endorsed having a very high participation level (34.6%) and 51 endorsed a high participation level (65.4%). In the Low Participation group, 33 endorsed a neutral participation level (73.3%), nine endorsed a low participation level (20.0%), and three endorsed a very low participation level (6.7%). There was a strong association between high family participation and loved one’s encouragement of their family to participate (*X*^2^_(1)_ = 4.29, *p* = 0.038). More specifically, 61.5% of the High Participation group indicated that their loved one encourages their participation in their care, whereas only 42.2% of the Low Participation group reported being encouraged to participate by their loved one.

**Table 2:** Sociodemographic information for family members

	Low participation group (*N* = 45)	High participation group (*N* = 78)	Full sample *(N* = 123)
Age			
< 40 years old	11 (24.4)	11 (14.1)	22 (17.9)
40–49 years old	15 (33.3)	21 (26.9)	36 (29.3)
50–59 years old	14 (31.1)	34 (43.6)	48 (39.0)
≥ 60 years old	5 (11.1)	9 (11.5)	14 (11.4)
Not reported	0 (0.0)	3 (3.8)	3 (2.4)
Gender			
Male *N* (%)	2 (4.4)	12 (15.4)	14 (11.4)
Female *N* (%)	42 (93.3)	64 (82.1)	106 (86.2)
Other gender identity/Not reported *N* (%)	1 (2.2)	2 (2.6)	3 (2.4)
Race			
American Indian/Alaska Native *N* (%)	0 (0.0)	0 (0.0)	0 (0.0)
Asian *N* (%)	8 (17.8)	13 (16.7)	21 (17.1)
Black/African American *N* (%)	12 (26.7)	15 (19.2)	27 (22.0)
Native Hawaiian/Other Pacific Islander *N* (%)	0 (0.0)	1 (1.3)	1 (0.8)
White *N* (%)	14 (31.1)	32 (41.0)	46 (37.4)
Other/not reported *N* (%)	13 (28.9)	18 (23.1)	31 (25.2)
Ethnicity			
Hispanic/Latino/Spanish Origin *N* (%)	13 (28.9)	19 (24.4)	32 (26.0)
Not Hispanic/Latino/Spanish Origin *N* (%)	32 (71.1)	56 (71.8)	88 (71.5)
Not reported *N* (%)	0 (0.0)	3 (3.8)	3 (2.4)
Educational level			
Some high school/High school diploma/GED *N* (%)	12 (26.7)	18 (23.1)	30 (24.4)
Some college/Associate’s or other 2-year degree/Bachelor’s degree *N* (%)	22 (48.9)	34 (43.6)	56 (45.5)
Some graduate school/Graduate or professional degree *N* (%)	10 (22.2)	22 (28.2)	32 (26.0)
Not reported *N* (%)	1 (2.2)	4 (5.1)	5 (40.7)
Relationship to loved one receiving care			
Parent *N* (%)	38 (84.4)	66 (84.6)	104 (84.6)
Other *N* (%)	6 (13.3)	9 (11.5)	15 (12.2)
Not reported *N* (%)	1 (2.2)	3 (3.8)	4 (3.25)
Age of loved one receiving care			
< 18 years old *N* (%)	4 (8.9)	6 (7.7)	10 (8.1)
18–21 years old *N* (%)	17 (37.8)	31 (39.7)	48 (39.0)
22–25 years old *N* (%)	17 (37.8)	21 (26.9)	38 (30.9)
≥ 26 years old *N* (%)	6 (13.3)	17 (21.8)	23 (18.7)
Not reported *N* (%)	1 (2.2)	3 (3.8)	4 (3.25)

Then, using loved one’s encouragement of family participation as a covariate, results demonstrated that total SDM score was significantly higher in the High Participation group (*M* = 13.9, *SD* = 1.7) than in the Low Participation group (*M* = 11.6, *SD* = 3.3), *F*(1, 120) = 21.3, *p* < 0.001, *partial η*^*2*^ = 0.15. On the item level, significantly more participants in the High Participation group than in the Low Participation group endorsed that the team presented several ways to address an identified problem and inquired about individual and family preferences (*p* = 0.004), that they explained the pros and cons of care options, including taking no action (*p* = 0.036), that they explored family expectations and ideas about how problems are to be managed (*p* = 0.006), and they understood, appreciated, and respected family values, presenting options for care that were in line with those values (*p* = 0.047). No differences were found between groups on endorsements of the team asking about how much the family preferred to be involved and respecting or valuing family involvement (*p* = 0.190; Table 2).

## Discussion

The purpose of this study was to examine SDM as a tool of engagement for family members during the care experience of adolescents and young adults with FEP. While SDM has been identified as a promising communication tool in patient-center healthcare approaches, it remains not fully explored and implemented in settings with patients with severe mental health disorders (Zisman-Ilani et al., 2021a, 2021b). Consistent with prior literature indicating that SDM may be an engagement facilitator for individual with severe mental health symptoms, the significantly higher endorsement of SDM characteristics occurred among the High Participation of OTNY group (Deegan & Drake, 2006; Thomas et al., 2021). Additionally, SDM endorsement was found to be impacted by the attitudes of loved ones toward their family’s participation in mental health care.

Several limitations should be noted. First, our response rate was low (19.57%), which likely biased toward already engaged families. Second, our sample was likely biased towards endorsing a shared decision participatory model because those who responded already had high engagement levels. Third, the study was conducted in one state, so generalizations to other states are not supported. Fourth, this is a cross-sectional analysis, so interpretations of trends over time due to participation in OTNY cannot be made. Fifth, families in the High Participation group might be more likely to observe shared decision-making conversations, potentially contributing to the relationship found between the dichotomous participation variable and SDM items. Sixth, information was not captured for families that did not speak English, Spanish, or Mandarin, and these families may have had unique engagement needs related to linguistic and cultural backgrounds. Future research would benefit from including the patient’s gender, race, and ethnicity to clarify which patients are being represented in the family sample. Lastly, future evaluations should include patient’s perception of encouraging a family member to participate, which was not reported in this study.

In examining the therapeutic context in which SDM can be implemented, cultural considerations impacting communication, therapeutic alliance, and engagement should be considered. For example, the matching of race or cultural and ethnic group between clients and providers could impact the therapeutic alliance, suggesting an area for further SDM-related research (Oluwoye, 2023). Our data surrounding OTNY and SDM was not sufficiently nuanced to address these components. Additionally, the demographics of our sample differed from OTNY demographics most recently reported in 2016 (Bello et al.) and included fewer Black clients (22 vs. 39%). Further, the higher number of Black family members noted in the Low Participation group highlights potential limitations in understanding factors impacting the engagement of historically minoritized and marginalized groups (Oluwoye, 2021). Barriers to using SDM in this therapeutic context may include effective communication challenges and entrenched hierarchical, knowledge-based power dynamics (Pavlo et al., 2019).

Nevertheless, these findings lend cautiously optimistic support to SDM as a valuable tool in facilitating treatment engagement of family members of young adults with severe mental health symptoms, especially among families whose loved one’s support their family’s engagement in mental health services. When a loved one and their family members do not share an understanding of their experiences, this can create significant barriers for family engagement strategies. By examining whether a loved one encourages and supports their family involvement, this study contributes to knowledge about ways to personalize family involvement approaches. Future research is needed to investigate ways to tailor SDM to families with different levels of engagement and for families where a loved one’s encouragement is weak or absent. There is limited understanding of the mechanisms underlying family engagement in services for adolescents and young adults with FEP. Future research should also investigate ways to eliminate barriers, including inequities in access, to ensure full family engagement in services for all families.
